# Prevalence and Determinants of Susceptibility to Tobacco Smoking Among Students in The Gambia

**DOI:** 10.1093/ntr/nty128

**Published:** 2018-06-20

**Authors:** Isatou K Jallow, John Britton, Tessa Langley

**Affiliations:** 1 UK Centre for Tobacco and Alcohol Studies, Division of Epidemiology and Public Health, University of Nottingham, Nottingham, UK; 2 National Public Health Laboratories, Ministry of Health & Social Welfare (MoH&SW), Banjul, The Gambia

## Abstract

**Introduction:**

Smoking is the biggest preventable cause of death and kills about seven million people annually. As smoking prevalence is falling in developed countries, tobacco businesses are turning to low- and middle-income countries (LMICs) to generate new tobacco markets. To prevent young people from initiating smoking and becoming regular smokers, it is important to understand the causes of susceptibility to smoking. In this study, we report a nationwide survey of the prevalence and risk factors of smoking susceptibility among students aged 12–20 years in The Gambia.

**Methods:**

We used two-stage cluster random sampling to select students in secondary schools throughout The Gambia and questionnaire to collect data on demographic characteristics and indicators on susceptibility to initiating smoking.

**Results:**

Among the total sample of 10289 students, 9831 (96%; 55.6% girls and 44.4% boys, aged 12–20 years) nonsmokers were included in the analysis. Of these, 3333 (33.9%) were found to be susceptible to smoking. Smoking susceptibility was more common among students attending grant-aided schools, non-Muslims, who had smoking allowed at home, had family members or friends who smoke, were sent to purchase cigarettes, had poor knowledge of the harmful effects of smoking, noticed point-of-sale tobacco advertisements, and who had positive attitudes towards smoking.

**Conclusions:**

This study shows that susceptibility to smoking is common among students and associated with preventable exposures. Although based on cross-sectional data, these findings suggest that raising students’ awareness of the harmful effects of smoking and reducing the prevalence of adult smoking, extending tobacco advertising restrictions to include point-of-sale, are all important to preventing the uptake of smoking among students.

**Implications:**

This is the first study to provide detailed data on smoking susceptibility and risk factors in a nationally representative sample of young people in The Gambia. Our findings show that susceptibility to smoking is relatively high and associated with preventable measures. Our results also identify an urgent need to broaden the ban on tobacco advertising to explicitly include point-of-sale advertisements. These findings provide valuable information for tobacco control policies and evidence to enable targeted intervention for young people most at risk of initiating smoking.

## Introduction

Smoking kills about seven million people annually and is therefore one of the biggest avoidable causes of ill health in the world.^1^ As smoking prevalence is falling in developed countries, tobacco companies are turning to low- and middle-income countries (LMICs) to generate new growth in tobacco sales.^2,3^ Such countries represent an attractive market as young people represent the majority of the population.^4^ Smoking susceptibility has been found to be a strong predictor of smoking experimentation and young people who are susceptible to smoking have been identified to have double the risk of taking up smoking compared with those who are not susceptible.^5–7^ A number of factors that influence susceptibility to smoking initiation among young people have been identified in the literature. These include sociodemographic, environmental, socioeconomic, and behavioral characteristics.^6,8–10^ Understanding the factors that influence never smokers to initiate smoking is critical to shaping future smoking prevention program.^6,11^ However, data on risk factors for and their effects on susceptibility to smoking among young people in the world’s poorest nations, particularly in Sub-Saharan Africa, remain sparse.

In The Gambia, data on tobacco use among young people are limited. In a recent study of a nationally representative sample of Gambian students aged 12–20 years, we reported a prevalence of current smoking of 7.9% in boys and 1.5% in girls and found that shisha use was becoming increasingly popular.^12^ To our knowledge, data on smoking susceptibility in The Gambia are not available. Therefore, in this study, we report a nationwide survey of the prevalence of smoking susceptibility and the risk factors for susceptibility among young people in The Gambia.

## Methods

### Study Population and Variables

The study was carried out in a sample of Upper Basic Schools (UBS) and Senior Secondary Schools (SSS) throughout The Gambia, using methods described previously.^12^ Briefly, two-stage cluster sampling was used to generate a nationally representative sample of students in grades 7 to 12 (aged 12–20 years). In the first stage, schools were randomly selected from a list of schools with a probability proportional to their enrolment size and, in the second stage, classes within the selected schools were randomly selected from the total number of classes in the schools. All students in the selected classes were eligible to participate.

Participating students completed a self-administered questionnaire adapted from the WHO Global Youth Tobacco Survey (GYTS) questionnaire. Data were collected on a range of variables including demographic details, smoking susceptibility, exposure to tobacco advertisements and promotion, anti-smoking media messages, beliefs about the danger of smoking, and the perceived benefits of smoking. The questionnaire also included a series of questions on several indicators of tobacco use, exposure to second-hand smoke (SHS), support for public smoking regulations, and knowledge of the harmful effect of SHS; these data have been reported in a separate publication.^12^

We measured the outcome variable, susceptibility to smoking, using two standard GYTS questions: (1) “If one of your best friends offered you a tobacco product, would you smoke it?” and (2) “At any time during the next 12 months do you think you will smoke any form of tobacco?” Students who were currently nonsmokers and answered “definitely not” to both questions were coded as nonsusceptible and others who answered “probably not,” “probably yes,” or “definitely yes” to either question were labelled as susceptible to smoking.^13–15^ Current smoking was defined as any smoking of cigarettes, cigars, or pipes at any time in the past 30 days. All students who responded “definitely not” and “probably not” to the question on knowledge of the harmful effects of smoking were defined as having poor knowledge and those who responded “probably yes” and “definitely yes” were defined as having good knowledge. Ethical approvals for the survey were obtained from The Gambia Government/Medical Research Council (MRC) Joint Ethics Committee (SCC 1468v2) and from the Ethics Committee of the Faculty of School of Medicine and Health Sciences of the University of Nottingham, UK (OVS24022016 SoM EPH).

### Statistical Analysis

The data were initially entered in to Microsoft Access and exported to Stata version 15 for analysis and only nonsmokers were included in the analysis of the study. Descriptive and chi-square analyses were used to obtain estimates of the prevalence of susceptibility to smoke and to determine the association of smoking susceptibility to students’ demographic characteristics, awareness of tobacco advertisements, attitudes, beliefs, and perceived benefits of smoking. Univariate logistic regression was carried out first to look for association between smoking susceptibility (outcome variable) and the exposure variables. The exposure variables included gender, age, school locality, school funding source, religion, home smoking rules, family and friends’ smoking status, whether students were sent to purchase cigarettes for others, knowledge of the harmful effects of smoking, and exposure to tobacco advertisements. Gender, purchasing cigarettes for others, knowledge of the harmful effects of smoking, and exposure to tobacco advertisements were entered as binary variables and the rest of the exposure variables were categorical. We constructed a multivariate logistic regression model to ascertain the predicting factors of smoking susceptibility. Similar to previous analyses,^13,16^ we adjusted for a priori confounders comprising age, gender, and rural/urban area of school, which previous studies have suggested are associated with susceptibility to initiate tobacco use.^6,7^ Variables with *p* values equal to or less than 0.05 in the univariate analysis were included in the final model.

## Results

### Sample Description

Details of the socio-demographic characteristics and prevalence of active smoking in this survey population have been reported elsewhere.^12^ In brief, a total of 50 schools throughout the country participated in the study, including 33 upper basic and 17 senior secondary schools, comprising 13 private, 27 public, and 10 grant-aided schools. All schools (100%) approached during the study participated. A total of 10395 students were registered in the selected classes, of which 10289 (99%) students participated in the study. After excluding the 455 current smokers, 9831 students were included in the current analysis.

### Characteristics of the Study Population and Prevalence of Smoking Susceptibility

Detailed characteristics of the study participants by smoking susceptibility are summarized in Table 1. Among the total sample of 9831 students, 3333 (33.9%) of never smokers were susceptible to initiating smoking and 6498 (66.1%) were nonsusceptible. More than half (57.2%) of the participants were girls and 42.7% were boys. About half (51.5%) were aged between 14 and 17 years; 56.2% in UBS schools, 74.8% in public schools, and 76.2% of the students were attending schools in rural areas. The majority of the students were Muslims (96.2%), lived with their parents (80.0%), or lived in a home where smoking was not restricted (71.6%). Around 20% of students reported having a smoking parent and/or sibling. Most students had friends who were nonsmokers (67.5%) and about 43.7% were sent to buy cigarettes for their parents or others. Smoking susceptibility was more common among students attending grant-aided schools (45.3%), those of Christian or other faiths compared with Muslims, those who lived without parents (36.1%), who were subject to partial home smoking rules (42.5%), who had smoking mothers (63.1%), who had one or more family members and friends that smoked, and students who were sent to purchase cigarettes for others.

**Table 1.** Baseline Characteristics of the Study Participants by Smoking Susceptibility

Characteristics	Total *N* = 9831	Nonsusceptible *N* (%)	Susceptible *N* (%)	*p* value
	*N* (%)	6498 (66.1)	3333 (33.9)	
**Gender**				.628
Boys	4201 (42.7)	2788 (66.3)	1413 (33.6)	
Girls	5630 (57.2)	3710 (65.9)	1920 (34.1)	
**Age group**				.542
12–14	2167 (22.0)	1411 (65.1)	756 (34.8)	
15–17	5071 (51.5)	3369 (66.4)	1702 (33.5)	
18–20	2593 (26.3)	1781 (66.2)	875 (33.7)	
**School type**				.461
UBS	5533 (56.2)	3640 (65.7)	1893 (34.2)	
SSS	4298 (43.7)	2858 (66.5)	1440 (33.5)	
**School funding**				**<.001**
Public	7356 (74.8)	4946 (67.2)	2410 (32.7)	
Grant-aided	1015 (10.3)	555 (54.6)	460 (45.30)	
Private	1460 (14.8)	997 (68.20)	463 (31.70)	
**School locality**				.560
Rural	2335 (23.7)	1555 (66.6)	780 (33.4)	
Urban	7496 (76.2)	4943 (65.90)	2553 (34.0)	
**Religion**				<**.001**
Muslim	9463 (96.2)	6108 (66.6)	3055 (33.3)	
Christian	561 (5.7)	351 (62.5)	210 (37.4)	
Other	88 (0.8)	28 (31.8)	60 (68.1)	
**Living with parents**				**.021**
Yes	7873 (80.0)	5245 (66.6)	2625 (33.3)	
No	1953 (19.9)	1248 (63.9)	705 (36.1)	
**Home smoking rules**				<**.001**
No	7043 (71.6)	4799 (68.1)	2244 (31.8)	
Sometimes	1010 (10.2)	580 (57.4)	430 (42.5)	
Yes	1775 (18.0)	1117 (62.9)	658 (37.0)	
**Family smoking**				**<.001**
None	7125 (72.4)	4997 (70.1)	2128 (29.8)	
Mother	244 (2.4)	90 (36.8)	154 (63.1)	
Father	1110 (11.2)	657 (59.1)	453 (40.8)	
Brother/Sister	652 (6.6)	358 (54.9)	294 (45.0)	
Others	695 (7.0)	394 (56.6)	301 (43.3)	
**Number friends who smoke**				**<.001**
None	6640 (67.5)	4659 (70.1)	1981 (28.8)	
One	621 (6.3)	279 (44.9)	342 (55.0)	
Two	310 (3.1)	165 (53.2)	145 (46.7)	
Three or more	618 (6.2)	327 (52.9)	291 (47.0)	
Not sure	1633 (16.6)	1062 (66.1)	571 (34.9)	
**Sent to buy cigarettes for parents or others**				**<.001**
Yes	4298 (43.7)	2756 (64.1)	1542 (35.8)	
No	5523 (56.2)	3739 (67.7)	1784 (32.0)	
**Knowledge of harmful effects of smoking**				**<.001**
Good	4023 (40.9)	2295 (57.0)	1728 (42.9)	
Poor	5803 (59.0)	4203 (72.4)	1600 (27.5)	

*Chi-square test was used to calculate *p* value.

### Awareness of Tobacco Advertisement and Promotion

Participants’ awareness of tobacco advertisements and promotion, both overall and by smoking susceptibility status, is summarized in Table 2. About half (49.3%) of all students had noticed tobacco advertisements in the media (TV, videos, and movies) and one in six had noticed point-of-sale tobacco advertisements. Among those students who noticed point-of-sale tobacco advertisements, the brands most widely noticed were Bond Street (14.4%), Monte Carlo (6.1%), Piccadilly (5.9%), and Business Royal (2.6%; data not shown). About 12.9% of students had been offered a free cigarette by a tobacco company sales agent and 15.0% of participants owned an item with a tobacco logo or brand on it. The majority of students (58.8%) would not wear or use an item with a tobacco brand name or logo on it. More than half (59.7%) of all students indicated that they would support a tobacco advertisement ban. In addition, smoking susceptibility was more common among students who noticed point-of-sale tobacco advertisements (42.4%), who had been offered a free cigarette (56.9%), who owned an item with a tobacco brand or logo (51.9%), who were prepared to wear something with tobacco brand on it, and those who did not support a tobacco advertisement ban (43.1%).

**Table 2.** Awareness of Tobacco Advertisement and Promotion Among Study Participants

Characteristics	Total *N* = 9831	Nonsusceptible *N* (%)	Susceptible *N* (%)	*p* value
**Noticed tobacco advertisement in the media**				**.013**
No	4976 (50.6)	3231 (64.9)	1745 (35.0)	
Yes	4855 (49.3)	3269 (67.2)	1588 (32.7)	
**Noticed point-of-sale tobacco advertisement**				**<.001**
No	8251 (83.9)	5589 (67.7)	2662 (32.2)	
Yes	1580 (16.0)	909 (57.5)	671 (42.4)	
**Offered a free cigarette by tobacco company sales agents**				**<.001**
Yes	1275 (12.9)	549 (43.0)	726 (56.9)	
No	8540 (87.0)	5949 (69.6)	2591 (30.3)	
**Own anything with a tobacco brand/logo**				**<.001**
Yes	1470 (15.0)	707 (48.1)	763 (51.9)	
No	8332 (85.0)	5780 (69.3)	2552 (30.6)	
**Wear or use something with a tobacco brand**				**<.001**
Yes	1098 (11.9)	646 (56.1)	482 (43.9)	
May be	2938 (29.9)	1711 (58.2)	1227 (41.76)	
No	5775 (58.8)	4168 (72.1)	1607 (27.8)	
**Support tobacco advertisement ban**				**<.001**
No	3951 (40.2)	2248 (56.9)	1703 (43.1)	
Yes	5857 (59.7)	4245 (72.4)	1612 (27.5)	

*Chi-square test was used to calculate *p* value.

### Attitudes, Beliefs, and Perceived Benefits of Smoking Among Study Participants

Table 3 outlines the study participants’ attitudes, beliefs, and perceived benefits of smoking by smoking susceptibility status.

**Table 3.** Attitudes, Beliefs, and Perceived Benefits of Smoking Among Study Participants

Characteristics	Total *N* = 9831	Nonsusceptible *N* (%)	Susceptible *N* (%)	*p* value
**Difficult to quit once smoking is initiated**				**<.001**
No	4140 (42.1)	3075 (74.2)	1065 (25.7)	
Yes	5691 (57.8)	3423 (60.1)	2268 (39.8)	
**Make people more comfortable in social gathering**				**<.001**
No	6296 (64.0)	4300 (68.3)	1996 (31.7)	
Yes	1475 (15.0)	805 (54.5)	670 (45.4)	
Don’t know	2055 (20.9)	1393 (67.7)	662 (32.2)	
**Safe to smoke tobacco for only a year or two as long as you quit after that**				**<.001**
No	6376 (64.8)	4920 (77.1)	1456 (22.8)	
Yes	3455 (35.1)	1578 (45.6)	1877 (54.3)	
**Have more or less friends**				**<.001**
Less friends	4728 (48.1)	3425 (72.4)	1303 (27.5)	
More friends	2188 (22.2)	1309 (59.8)	879 (40.1)	
No difference	2898 (29.5)	1762 (60.8)	1136 (39.2)	
**Make people more or less attractive**				**<.001**
Less attractive	5470 (55.6)	3924 (71.7)	1546 (28.2)	
More attractive	1756 (17.8)	1079 (61.4)	677 (38.5)	
No difference to nonsmokers	2597 (26.4)	1493 (57.4)	1104 (42.5)	
**Can improve general health status**				**<.001**
Yes	943 (9.6)	436 (46.2)	507 (53.7)	
No	6993 (71.1)	4988 (71.3)	2005 (28.6)	
Don’t know	1888 (19.2)	1074 (56.8)	814 (43.1)	
**Can help to lose weight**				**<.001**
Yes	4621 (47.0)	3287 (71.3)	1334 (28.8)	
No	3041 (30.9)	1811 (59.5)	1230 (40.4)	
Don’t know	2164 (22.0)	1400 (64.7)	764 (35.3)	
**Can make people more relaxed**				**<.001**
Yes	1850 (18.8)	1051 (56.8)	799 (43.1)	
No	5374 (54.6)	3645 (67.8)	1729 (32.1)	
I don’t know	2602 (26.4)	1802 (69.2)	800 (30.7)	

*Chi-square test was used to calculate *p* value.

The majority (57.8%) of the students agreed that it would be difficult to quit smoking once initiated. Around 15.0% of the participants believed that smoking makes people more comfortable at social gatherings and 35.1% that it is safe to smoke tobacco as long as you can quit later. One in five students also believed that smoking can help people to have more friends. In addition, about one in six participants believed that smoking makes people more attractive (17.8%); one in ten participants believed that smoking can help improve general health status; almost half (47.0%) believed that smoking can help people lose weight; and 18.8% believed that smoking makes people feel more relaxed. Perceptions that smokers are more comfortable at social gatherings and are more attractive and relaxed than nonsmokers were significantly positively associated with susceptibility to smoking. Additionally, susceptibility to smoking was more common among students who believed that it is safe to smoke so long as you quit later, those who believed that smoking can help to lose weight, and those who believed that smoking can improve general health status.

### Independent Determinants of Smoking Susceptibility Among the Study Participants

The association between smoking susceptibility and student characteristics is outlined in Table 4. After adjusting for all independent variables, smoking susceptibility was more common among students attending grant-aided schools (OR = 1.59, 95% CI = 1.35 to 1.87), of Christian or other non-Muslim faiths (OR = 2.01, 95% CI = 1.17 to 3.46), who had smoking sometimes allowed in their homes (OR = 1.33, 95% CI = 1.13 to 1.56), had family members or friends who smoked, and were sent to purchase cigarettes for others. Additionally, students who had poor knowledge of the harmful effects of smoking (OR = 1.65, 95% CI = 1.48 to 1.83) and those who noticed point-of-sale tobacco advertisements (OR = 1.15, 95% CI = 1.01 to 1.32) were significantly more likely to be susceptible to smoking. Students who had been offered a free cigarette, owned and used a tobacco branded item, who believed that it is difficult to quit once smoking is initiated, that it is safe to smoke as long as one quits later on, that smoking can improve general health status, and that smoking can make people relaxed were significantly more likely to be susceptible to smoking. Students with perceptions that smokers have more friends, being more attractive, and that smoking can help to lose weight were significantly less likely to be susceptibility to smoking.

**Table 4.** Prevalence and Determinants of Susceptibility to Smoking Among Current Nonsmokers

Characteristics	Total *N* = 9831	Susceptible nonsmokers *N* = 3333	Unadjusted OR	*p* value	Adjusted OR	*p* value
**Gender**				.628		.585
Boys	4201	1413 (33.6)	1		1	
Girls	5630	1920 (34.1)	1.02 (0.93–1.11)		1.02 (0.92–1.13)	
**Age group**				.541		.280
12–14	2167	756 (34.8)	1		1	
15–17	5071	1702 (33.5)	0.94 (0.84–1.04)		0.97 (0.86–1.10)	
18–20	2593	875 (33.7)	0.95 (0.84–1.07)		1.07 (0.92–1.23)	
**School funding**				<.001		<.001
Public	7356	2410 (32.7)	1		1	
Grant-aided	1015	460 (45.30)	1.70 (1.48–1.94)		1.59 (1.35–1.87)	
Private	1460	463 (31.70)	0.95 (0.84–1.07)		1.04 (0.90–1.20)	
**School locality**				.560		.477
Rural	2335	780 (33.4)	1		1	
Urban	7496	2553 (34.0)	1.02 (0.93–1.13)		1.04 (0.92–1.17)	
**Religion**				<.001	1	.004
Muslim	9463	3055 (33.3)	1		0.99 (0.80–1.22)	
Christian	561	210 (37.4)	1.19 (1.00–1.42)		2.01(1.17–	
Other	88	60 (68.1)	4.28 (2.72–6.72)		3.46)	
Living with parents				.021		.291
Yes	7873	2625 (33.3)	1		1	
No	1953	705 (36.1)	0.88 (0.79–098)		1.06 (0.94–1.20)	
**Home smoking rules**				<.001		.001
No	7043	2244 (31.8)	1		1	
Sometimes	1010	430 (42.5)	1.58 (1.38–1.81)		1.33 (1.13–1.56)	
Yes	1775	658 (37.0)	1.25 (1.13–1.40)		0.98 (0.86–1.12)	
**Family smoking**				<.001		<.001
None	7125	2128 (29.8)	1		1	
Mother	244	154 (63.1)	4.01 (3.08–5.23)		2.56 (1.87–3.50)	
Father	1110	453 (40.8)	1.61 (1.42–1.84)		1.48 (1.26–1.74)	
Brother/Sister	652	294 (45.0)	1.92 (1.63–2.26)		1.91 (1.58–2.31)	
Others	695	301 (43.3)	1.79 (1.53–2.10)		1.50 (1.24–1.81)	
**Number of friends who smoke**				<.001		<.001
None	6640	1981 (28.8)	1		1	
One	621	342 (55.0)	2.88 (2.44–3.40)		1.62 (1.33–1.99)	
Two	310	145 (46.7)	2.06 (1.64–2.59)		1.28 (0.97–1.68)	
Three or more	618	291 (47.0)	2.09 (1.77–2.47)		1.48 (1.21–1.81)	
Not sure	1633	571 (34.9)	1.26 (1.12–1.41)		1.06 (0.93–1.21)	
**Sent to buy cigarettes for others**				<.001		<.001
Yes	4298	1542 (35.8)	1		1	
No	5523	1784 (32.00)	0.85 (0.78–0.92)		0.81 (0.73–0.90)	
**Knowledge of harmful effects of smoking**				<.001		<.001
Good	4023	1728 (27.5)	1		1	
Poor	5803	1600 (42.9)	1.97 (1.81–2.15)		1.65 (1.48–1.83)	
**Noticed tobacco advertisement in media**				.013		.148
No	4976	1745 (35.07)	1		1	
Yes	4855	3231 (64.9)	0.90 (0.82–0.97)		0.92 (0.84–1.02)	
**Noticed point of sale tobacco advertisement**				<.001		.032
No	8251	2662 (32.2)	1		1	
Yes	1580	671 (42.4)	1.54 (1.38–1.72)		1.15 (1.01–1.32)	
**Offered a free cigarette by tobacco company sales agents**				<.001		<.001
Yes	1275 (12.9)	726 (56.9)	1		1	
No	8540 (87.0)	2591 (30.3)	0.32 (0.29–0.37)		0.50(0.43–0.57)	
**Own anything with a tobacco brand/logo**				<.001		<.001
Yes	1470 (15.0)	763 (51.9)	1		1	
No	8332 (85.0)	2552 (30.6)	0.40 (0.36–0.45)		0.76 (0.66–0.87)	
**Wear or use something with a tobacco brand**				<.001		<.001
Yes	1098 (11.9)	482 (43.9)	1		1	
May be	2938 (29.9)	1227 (41.76)	0.91 (0.79–1.05)		1.03 (0.88–1.22)	
No	5775 (58.8)	1607 (27.8)	0.49 (0.43–0.56)		0.70 (0.59–0.82)	
**Difficult to quit once smoking is initiated**				**<.001**		**<.001**
No	4140 (42.1)	1065 (25.7)	1		1	
Yes	5691 (57.8)	2268 (39.8)	1.91 (1.75–2.08)		2.01 (1.81–2.22)	
**Make people more comfortable in social gathering**				.002		.424
No	6296 (64.0)	1996 (31.7)	1		1	
Yes	1475 (15.0)	670 (45.4)	0.78 (0.70–0.88)		0.93 (0.81–1.08)	
Don’t know	2055 (20.9)	662 (32.2)	0.80 (0.71–0.90)		0.88 (0.73–1.08)	
**Safe to smoke tobacco for only a year or two as long as you quit after that**				<.001		**<.001**
No	6376 (64.8)	1456 (22.8)	1		1	
Yes	3455 (35.1)	1877 (54.3)	4.01 (3.67–4.39)		3.35 (3.04–3.70)	
**Have more or less friends**				<.001		**<.001**
Less friends	4728 (48.1)	1303 (27.5)	1		1	
More friends	2188 (22.2)	879 (40.1)	0.56 (0.50–0.63)		0.69 (0.60–0.78)	
No difference	2898 (29.5)	1136 (39.2)	0.96 (0.85–1.07)		0.85 (0.74–0.97)	
**Make people more or less attractive**				<.001		**<.001**
Less attractive	5470 (55.6)	1546 (28.2)	1		1	
More attractive	1756 (17.8)	677 (38.5)	0.62 (0.56–0.70)		0.89 (0.78–1.02)	
No difference	2597 (26.4)	1104 (42.5)	1.17 (1.04–1.33)		1.29 (1.11–1.51)	
**Can improve general health status**				<.001		**<.001**
Yes	943 (9.6)	507 (53.7)	1		1	
No	6993 (71.1)	2005 (28.6)	0.34 (0.30–0.39)		0.50 (0.43–0.59)	
Don’t know	1888 (19.2)	814 (43.1)	0.65 (0.55–0.76)		0.74 (0.61–0.89)	
**Can help to lose weight**				<.001		**<.001**
Yes	4621 (47.0)	1334 (28.8)	1		1	
No	3041 (30.9)	1230 (40.4)	1.67 (1.51–1.84)		1.40 (1.25–1.58)	
Don’t know	2164 (22.0)	764 (35.3)	1.34 (1.20–1.49)		1.22 (1.06–1.41)	
**Can make people more relaxed**				<.001		**<.001**
Yes	1850 (18.8)	799 (43.1)	1		1	
No	5374 (54.6)	1729 (32.1)	0.62 (0.55–0.69)		0.69 (0.61–0.79)	
I don’t know	2602 (26.4)	800 (30.7)	0.58 (0.51–0.66)		0.56 (0.48–0.66)	

## Discussion

This is the first study to provide detailed data on smoking susceptibility and risk factors in a nationally representative sample of adolescent school students in The Gambia. We found that one in three students were susceptible to smoking. Susceptibility was more common among students attending grant-aided schools and non-Muslims. Young people in our sample were more likely to be susceptible to smoking if they had smoking allowed in their homes, had family or friends who smoked, were sent to purchase cigarettes for others, had poor knowledge of the harmful effect of smoking, and noticed tobacco at the point-of-sale. Additionally, positive attitudes, beliefs, and perceived benefits of smoking were significantly associated with susceptibility to smoking. Our study was cross sectional and has limited ability to attribute causality to smoking susceptibility. All estimates in our assessment were based on self-reports which might be affected by reporting bias. Also, although smoking susceptibility has been shown consistently to be a risk factor for smoking experimentation,^7,17,18^ its predictive value for sustained future smoking is limited. Given the number of variables included in the analysis, we acknowledge the possibility of type I error given the fact that included variables may be highly correlated. Our survey was conducted in schools and therefore may not be representative of Gambian youth as a whole. Data from the Ministry of Education indicate gross enrolment rates of 68.12% and 41.2% for UBS and SSS, respectively^19^; this suggests that our data are more representative for among younger age groups. However, doing surveys in schools is one of the most efficient ways to collect data among young people and our study has provided very useful data on a topic with very sparse information particularly in Sub-Saharan Africa.

Previous studies on smoking initiation among young people in The Gambia and in West Africa are limited. However, our finding that one in three students were susceptible to smoking initiation is consistent with work from other developing and developed countries.^15,20^ Moreover, young people in other countries in Africa at a similar stage of economic development to The Gambia are likely to be exposed to similar risk factors and we think it is likely that our findings will be generalizable to such countries. Given the validation of smoking susceptibility as a predictor of smoking experimentation,^7,18^ these findings suggest that smoking prevalence among young people is likely to rise in the near future in The Gambia. This is particularly important in many Sub-Saharan African countries with low current-smoking rates but at high risk of the smoking epidemic.

Our finding that susceptibility to smoking varies significantly between types of schools and that religious beliefs influence smoking susceptibility is consistent with existing evidence.^6,15,20,21^ The link between susceptibility to smoking with socio-cultural factors, and particularly religious faith and attending nonpublic schools is consistent with our previous findings^12^ that non-Muslim students attending grant-aided or private schools were more likely to be current smokers. We found that students who live in homes with only some smoking restrictions were more likely to be susceptible to smoking. This finding is in line with previous studies reporting that the absence of, or a partial ban on home smoking, are associated with an increased risk of smoking susceptibility^22–24^ and that partial home smoking bans have not been effective in preventing smoking initiation.^23,25^

In line with existing findings,^13–15^ we found that students who had parents or friends who smoked and had been sent to purchase cigarettes for their parents or other older adults were significantly more likely to be susceptible to smoking initiation. These characteristics appear to identify contact with others who smoke, and these findings indicate that efforts to minimize parents and peer smoking are needed. Students’ knowledge of the harmful effects of smoking as they relate to susceptibility to initiate smoking is well-documented.^13,15,26^ Our results provide further confirmation that having good knowledge about the harmful effects of smoking serves as a protective factor against susceptibility to initiate smoking and underscores the importance of education on the harmful effects of smoking.

The finding that awareness of point-of-sale tobacco advertising is associated with smoking susceptibility is consistent with previous studies in both developing and developed countries.^27–30^ Given that all forms of tobacco advertisements have been banned in The Gambia since 2003, our findings demonstrate worryingly high levels of exposure to tobacco advertisements, which may be a reflection of poor implementation of the Tobacco Advertisement Act. To reduce exposure to tobacco advertisements and promotion, the ban needs to be comprehensive.^29,31^ Advertisements were predominantly seen on television, movies, magazines, radio, and on the internet, which are available online and from broadcasters based within The Gambia and outside. It is possible that media advertisements within and outside the country are not adequately regulated and subjected to the advertisement ban.^32,33^

Although we did not find exposure to anti-tobacco media messages to be a significant predictor of susceptibility to smoking in our study, we found that more than half of all students did not hear or see any anti-tobacco media messages in the past 30 days preceding this study. This suggests that messages are insufficient and even available messages are not delivered effectively. This highlights the need for more mass media campaigns and it is also important that anti-smoking media messages are appropriately delivered without interference by tobacco companies.

Limited research is available to compare and explore young Gambians perspectives, attitudes and beliefs about smoking. However similar to previous findings elsewhere^13,34^, we found that students who had positive attitudes, beliefs, and perceived benefits of smoking were significantly susceptible to smoking. Preventive measures and efforts that particularly focus on various social and behavioral aspects are needed.

## Conclusion

This study has shown that susceptibility to smoking is relatively high among students in The Gambia. To help minimize future smoking initiation among young people, intervention may need to be targeted particularly at parents and peers who smoke and raising students’ awareness of the harmful effects of smoking. This may help to reduce future smoking among students and provide the maximum benefit as a protective factor against smoking initiation. Our findings also suggest that there is a need to broaden the ban on tobacco advertising to explicitly include all forms of media and point-of-sale advertisements. In addition, strict enforcement of the ban on tobacco advertisements should be a high priority for policy makers.

## Funding

This work was supported by the Medical Research Council [grant number MR/K023195/1], [RA08D9] through the UK Centre for Tobacco and Alcohol Studies (http://www.ukctas.net); and a scholarship funding from the Islamic Development Bank (IDB).

## Ethical approval

This research project was reviewed and approved by The Gambia Government/Medical Research Council (MRC) Joint Ethics Committee in The Gambia and the Research Ethics Committee of the Faculty of Medicine and Health Sciences, University of Nottingham, UK.

Provenance and peer review: externally peer reviewed.

## Declaration of Interests

*None declared*.
